# The social and family evaluation (SAFE) scale for caregivers of individuals with disorders of consciousness: preliminary results

**DOI:** 10.1007/s10072-024-07685-4

**Published:** 2024-07-27

**Authors:** F. G. Magnani, M. Cacciatore, F. Barbadoro, C. Ippoliti, D. Sattin, A. Magliacano, F. Draghi, A. De Nisco, B. Hakiki, F. Cecchi, M. Spinola, A. Estraneo, M. Leonardi

**Affiliations:** 1 SC Neurologia, Salute Pubblica, Disabilità, Fondazione IRCCS Istituto Neurologico Carlo Besta, via Celoria, Milan, Italy; 2 Istituti Clinici Scientifici Maugeri IRCCS, Health Directorate, Via Camaldoli 64, Milan, 20138 Italy; 3 IRCCS Fondazione Don Carlo Gnocchi ONLUS, Florence, Italy; 4 Department of Experimental and Clinical Medicine, University of Florence, Firenze, Italy

**Keywords:** Vegetative state, Minimally conscious state, Family members, Assessment, Telemedicine

## Abstract

**Background:**

Caregivers’ involvement in the diagnostic and monitoring processes of the level of consciousness of patients with Disorders of Consciousness (DoC) is strongly encouraged by international guidelines, as current literature suggests a better chance to detect behavioural responses when caregivers are involved in clinical assessments. Since caregivers’ involvement during clinical assessments can be difficult, the Social And Family Evaluation (SAFE) scale has been recently proposed as a standardised tool that caregivers can autonomously use to collect their opinions about the level of consciousness of patients with DoC, based on the behaviours manifested by the patients in a given time-window.

**Objective:**

Providing preliminary results concerning SAFE adoption.

**Methods:**

22 patients with DoC were assessed through the Coma Recovery Scale-revised (CRS-r), while their caregivers filled-in the SAFE.

**Results:**

The SAFE showed a very high internal consistency, very high test-retest reliability, and high criterion validity when correlated to the CRS-r total score. Moreover, in line with the literature, the SAFE allowed the detection of some behaviours indicative of a higher level of consciousness than those detected by clinicians through the CRS-r in more than half of the sample.

**Conclusion:**

Overall, these preliminary data are promising for the adoption of the SAFE to collect the opinions of the caregivers about the level of consciousness of patients with DoC, especially in those settings where it would be otherwise difficult to monitor the patients, such as long-term care structures and at home, as a tool for telemedicine allowing the monitoring of patients in remote settings.

**Supplementary Information:**

The online version contains supplementary material available at 10.1007/s10072-024-07685-4.

## Introduction

After severely acquired brain injuries, many patients may end up with a Disorder of Consciousness (DoC) encompassing different clinical conditions characterized by impairments of the self- and/or environmental awareness that can last more than 28 days from brain injury (i.e., the so-called prolonged DoC), and persists many years (i.e., chronic DoC) [1]. Taking care of these patients is of uttermost importance, and it cannot forego the correct diagnosis which has important consequences on the definition of prognosis, planning of the care pathways, and the adoption of tailored rehabilitative interventions [2]. Specifically, depending on the Level of Consciousness (LoC), DoC patients can be divided into Vegetative State (VS), also called Unresponsive Wakefulness Syndrome (UWS; [3]) when only the reflexive behaviours are present, and Minimally Conscious State (MCS) when reproducible, even if not consistent, behavioural responses are detected [4]. When patients show communication abilities and/or functional object use, they are considered emerging from MCS (eMCS).

To date, the DoC diagnosis relies on the Coma Recovery Scale-revised (CRS-r) consisting of six different subscales addressing arousal, auditory, visual, motor, oro-motor, and communication abilities by observing the patient’s responses to external stimulations by a professional examiner [5]. Despite the CRS-r being the gold standard to diagnose DoC [6], many factors could affect its reliability, such as the patient’s arousal fluctuations, pain, and the presence of sensory, cognitive, and motor impairments [7, 8]. To reduce the misdiagnosis risk, both the clinicians and the scientific community agree upon the adoption of multiple evaluations to define the patient’s best performance [9]. Moreover, well-established evidence already showed that the adoption of salient stimuli determines a higher probability of detecting patient responses (see [10] for a systematic review). Since informal caregivers represent salient stimuli for patients, they can contribute to the improvement of diagnostic accuracy in patients with DoC. Indeed, in the study by Formisano et al. [11] the total score at the CRS-r and Post Coma Scale was higher when the assessment was performed by the clinician in the presence of the informal caregiver, due to a higher score in the subscales requiring the stimuli administration by the caregiver. Similarly, other works reached the same results when the informal caregivers contributed actively to the stimuli administration [12, 13]. Furthermore, the involvement of informal caregivers during the patients’ assessment, management, and monitoring purposes is strongly encouraged by the Royal College of Physicians (RCP) guidelines [14] which suggest also using appropriate tools to record caregivers’ observations (RCP recommendation 2.5; [14]).

Nevertheless, the adoption of the above-mentioned solutions to reduce the misdiagnosis risk and improve the diagnostic accuracy in patients with DoC is not easily applicable in all settings, such as either long-term care structures where expert clinicians might not be able to perform daily clinical assessments, or at home where caregivers are often alone with the patients, thus preventing the possibility to appropriately monitor the patients’ LoC over time. Moreover, combining the presence of both clinicians and informal caregivers can be problematic also in acute and rehabilitation clinical centres, since caregivers might be not daily present, and they usually visit their family members at the end of their workday, whilst the clinical evaluations are usually performed earlier during the day [15]. On the other hand, as already pointed out by previous studies [15, 16], there is a lack of tailored instruments that informal caregivers can manage independently from clinicians to be formally involved in the assessment and monitoring procedure.

For these reasons, the Social And Family Evaluation (SAFE) scale has been recently developed representing an instrument that informal caregivers can use to inform clinicians about possible changes in the patient’s LoC, basing their opinions on the observed behaviours during the direct interactions with the patient in a given time frame (i.e., past 15 days) [15]. Specifically, SAFE is based on the CRS-r, including items referring to the same behaviours the clinicians rely on to perform the diagnosis of MCS and eMCS, in line with the recent simplified evaluation of consciousness disorders [17]. Specifically, in developing the SAFE, the CRS-r items indicative of voluntary behaviours (i.e., non-reflexive) have been reworded to be understandable by non-expert individuals such as informal caregivers. These items were arranged in 5 subscales addressing visual and sensory-motor domains, language/communication, responses to pain, and task execution. The items’ arrangement into slightly different subscales compared to the CRS-r was functional to a better understanding of the items themselves by non-expert individuals; however, each SAFE item has the equivalent one in the CRS-r (for more details on the SAFE developing process, see [15]), allowing the adoption of a common language between clinicians and informal caregivers. In this context, SAFE could be an important tool that would allow clinicians to monitor the patient through the informal caregivers’ assessment, also allowing the informal caregivers to align with the standard assessment derived from scientific knowledge.

Although the SAFE validation process is still ongoing, the present study aimed to present the preliminary results on a sample of 22 patients with DoC, both in post-acute and chronic phases.

## Materials and methods

### Participants

Twenty-two informal caregivers of patients with DoC were enrolled in one neurological institute and two neurorehabilitation centres for severe acquired brain injury according to the following inclusion criteria: (i) age ≥ 18 years; (ii) being the main informal caregiver, thus regularly spending time with the DoC patient and being aware of the clinical situation of the patient; (iii) being Italian speakers; (iv) having subscribed the informed consent form.

Moreover, the 22 patients with DoC the enrolled caregivers took care of were enrolled if they presented with the following inclusion criteria: (i) age ≥ 18 years; (ii) having a DoC diagnosis according to the CRS-r; (iii) time since injury ≥ 28 days; (iv) absence of previous neurological and psychiatric disorders; (v) subscription of the informed consent form by a legal representative.

The study was designed according to the ethical standards of the Declaration of Helsinki and received approval from the local Ethical Committees.

### Procedures

Sociodemographic (age, sex, education) and clinical (time since injury, aetiology) variables for each patient with DoC enrolled in the study were collected. Similarly, sociodemographic variables for each corresponding caregiver, namely the age, sex, education, and the weekly hours spent on average in caregiving activities were collected as well.

The Italian version of the CRS-r [18] was administered to each patient enrolled in the study by expert clinicians without the presence of the caregivers, multiple times during two consecutive weeks, and the best performance was considered to determine the LoC and the corresponding diagnosis, according to the international recommendations [1, 14, 19]. Both the CRS-r total score and subscore for each subscale were recorded and considered for data analyses.

After the CRS-r assessments, the corresponding informal caregiver, blind to the CRS-r evaluation, was asked to fill in the SAFE scale following a thoughtful explanation by the expert clinician. Specifically, the caregiver was asked to answer each SAFE item representing a certain behaviour for a specific sensory/cognitive domain, through a YES/NO answer by referring to the past 15 days. In other words, the caregiver had to express whether, in the past 15 days, the patient could be able to perform each specific behaviour included in the SAFE according to the same criteria derived from the CRS-r (see supplementary material for items and criteria of the Italian version of the SAFE scale). Two days after the first administration, each caregiver was asked to fill in again the SAFE scale by referring to the past 15 days. The choice of considering two days between the two SAFE administrations was functional to reduce the probability of any significant change in the patient’s LoC. The YES/NO answers for each SAFE administration were converted into 1/0 score for each item, representing the ability to perform or not a specific behaviour, respectively. By summing up all item scores, we computed the SAFE total score for both the first and the second SAFE administration that was considered for the analyses.

### Statistical analyses

Data were analysed using Jamovi (version 2.0.0.0; [20]).

Descriptive statistics were reported as either mean and standard deviation or median and interquartile ranges for continuous variables, and frequencies/percentages for nominal ones.

To evaluate the internal validity of the SAFE, we computed the Cronbach’s α by considering the items scores at the first SAFE administration. To assess for SAFE criterion validity, we performed a Spearman’s correlation analysis between the CRS-r and SAFE first administration total scores. Similarly, to account for test-retest reliability, we performed a Spearman’s correlation analysis between the SAFE total score obtained at the two different time points by the same informal caregivers.

Moreover, to test whether SAFE was able to discriminate between patients with different LoC, a Mann-Whitney test was conducted on the SAFE total score at the first administration comparing patients with DoC depending on their LoC according to CRS-r.

Finally, to better explore the caregivers’ tendencies in judging the presence/absence of voluntary/non-reflexive behaviours in patients with DoC, we qualitatively checked whether there was a correspondence between CRS-r and SAFE evaluations. Specifically, we considered the highest-level behaviour detected by the clinician in each CRS-r subscale, and we checked if the same behaviour was detected also by the informal caregiver through the SAFE and vice versa. Consequently, we determined the absence of concordance between CRS-r and SAFE for each patient with DoC if at least one behaviour was discordant between the two scales. Then, considering the discordant cases, we qualitatively checked if the behaviours detected through the SAFE would suggest a different LoC than CRS-r.

To further explore if there were any factors associated with caregivers’ tendencies in overestimating/underestimating patients’ LoC, we divided the entire sample into two groups depending on the discordance between CRS-r and SAFE (concordant vs. discordant cases), and we checked for any difference between them by considering caregivers’ sociodemographic variables (age, sex, education), hours of care, time since injury, and patients’ LoC according to CRS-r (VS/UWS vs. being at least MCS). These comparisons were performed by means of either independent sample t-test or Mann-Whitney test depending on whether the normality assumption was met or violated, respectively, according to the results at the Shapiro-Wilk normality test. Moreover, we used the Chi-squared test when considering nominal variables across the two groups.

The significance level was set at *p* < .05 for all the analyses.

## Results

The 22 enrolled caregivers (median age ± IQR: 61 ± 15.8; median education ± IQR: 11 ± 5; 15 females) took care of patient for 8.48 ± 4.83 h/week on average. The 22 corresponding DoC patients (median age ± IQR: 54 ± 24.3; median education ± IQR: 9 ± 5; 14 males) were characterized by a time since injury of 20.31 ± 28.02 months on average, with a prevalence of non-traumatic aetiology (77.3%) and VS/UWS diagnosis (59.1%). See Table 1 for further details on the entire sample of patients.

**Table 1:** Main features of the entire sample of patients by their diagnosis. The CRS-r and SAFE first administration total scores (median and Interquartile Range), Time since Injury in months (mean and standard deviation), aetiology divided into TBI and non-TBI (percentage), and the hours of care by the caregivers (mean and standard deviation) are reported

Diagnosis (%)	CRS-*r* total score (IQR)	SAFE total score (IQR)	TSI	Aetiology %		Hours of care
				*TBI*	*Non-TBI*	
*VS/UWS (59.1)*	5 (3)	0 (1)	23.4 ± 34.6	13.64	45.45	9.21 ± 5.09
*MCS- (18.2)*	10 (0.25)	2.5 (2)	12.7 ± 18.6	9.09	9.09	9 ± 5.77
*MCS+ (13.6)*	12 (1.5)	5 (2)	9.77 ± 8.72	0	13.63	7.33 ± 4.51
*eMCS (9.1)*	21.5 (0.5)	11 (2)	31 ± 3.7	0	9.09	4.75 ± 1.06

*Abbreviations* VS/UWS = Vegetative State/Unresponsive Wakefulness Syndrome; MCS-= Minimally Conscious State minus; MCS + = Minimally conscious State plus; eMCS = emerging from Minimally Conscious State; CRS-r = Coma Recovery Scale-revised; SAFE = Social And Family Evaluation; TSI = Time Since Injury; TBI = Traumatic Brain Injury

The SAFE scale showed a very high internal consistency (α = 0.912). Moreover, the SAFE showed high criterion validity when correlated to the CRS-r total score (Spearman’s rho = 0.708; *p* < .001), and a very high test-retest reliability (Spearman’s rho = 1; *p* < .001).

**Fig. 1:**
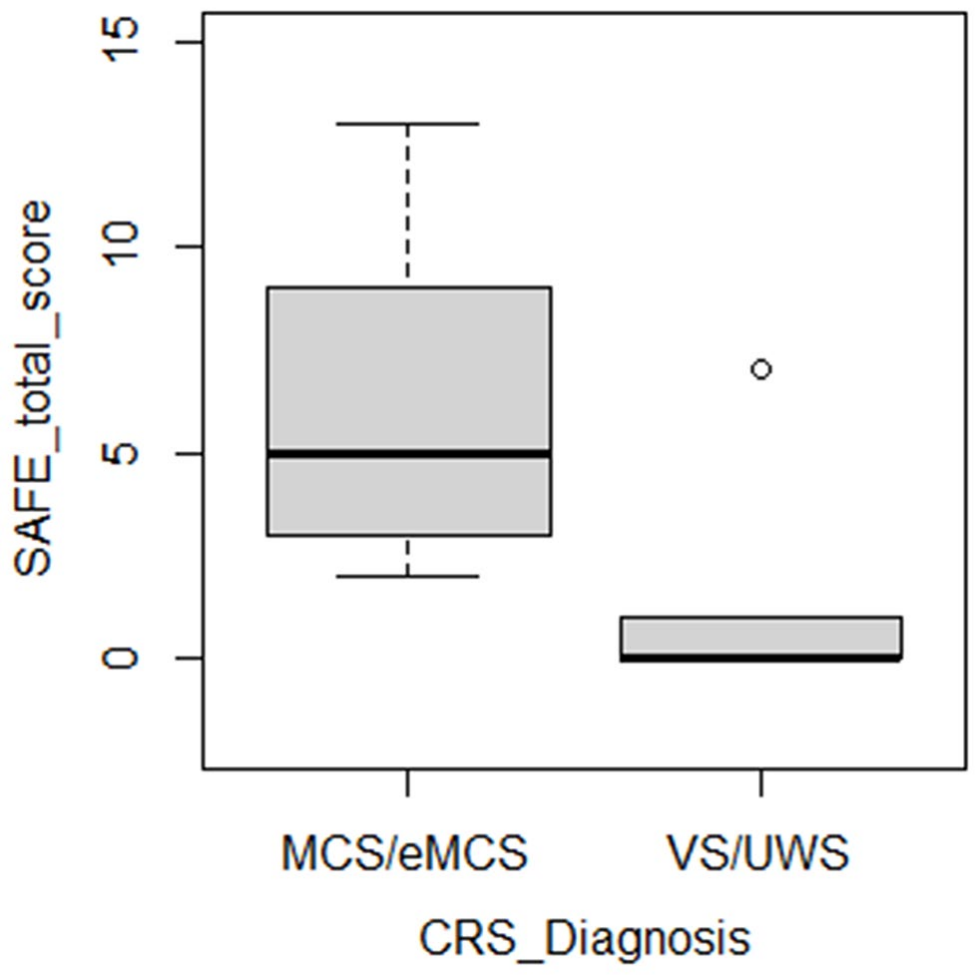
SAFE total score distribution across diagnoses. The box-plots represent the total score at the SAFE first administration in both VS/UWS and (at least) MCS patients according to the CRS-r diagnosis. *Abbreviations* SAFE = Social And Family Evaluation Scale; CRS-r = Coma Recovery Scale-revised; VS/UWS = Vegetative State/Unresponsive Wakefulness Syndrome; MCS = Minimally Conscious State; eMCS = emerging from Minimally Conscious State

Since only two patients with DoC were diagnosed as eMCS according to CRS-r, we divided the patients’ sample into two groups depending on whether they were diagnosed as either VS/UWS (*n* = 13) or at least MCS (i.e., showing voluntary/non-reflexive responses; *n* = 9) at the clinical assessment with the CRS-r to explore the SAFE ability to discriminate between them. We found a significant difference between the two groups when considering the SAFE total score at the first assessment (U = 5.50; *p* < .001; Fig. 1). Specifically, the patients showing at least minimal signs of consciousness at the CRS-r had a higher SAFE total score (median ± IQR = 5 ± 6) than UWS patients (median ± IQR = 0 ± 1).

By exploring caregivers’ tendencies in judging the presence/absence of voluntary/non-reflexive behaviours through the SAFE, we found a correspondence between the behaviours detected through the CRS-r and SAFE in 10 patients out of 22. Although the SAFE always detected at least one higher-level behaviour than CRS-r in all the 12 discordant cases, the reverse (i.e., higher-level behaviour detected by the CRS-r than SAFE) was true for three different single behaviours in 3 out of 12 cases (see Table 2 and supplementary table S1 for more details on single behaviours discordance). Qualitatively, the visual pursuit and the reproducible movement to command were the behaviours most frequently detected by the informal caregivers and not by the clinicians, whereas no behaviours were subjected to significant underestimation by the caregivers (see supplementary table S1). By considering the behaviours detected through the SAFE in the 12 discordant cases, a different LoC might be hypothesized in 8 cases compared to what was derived from the CRS-r (see Table 2).

**Table 2:** Discordant cases between SAFE and CRS-r. SAFE and CRS-r total scores (second and fourth column, respectively) as well as the corresponding LoC (third and fifth column, respectively) are reported. In the last two columns, the behaviours detected through the SAFE over the CRS-r and vice versa are reported. Asterisk highlights the behaviours suggestive of a different LoC according to SAFE; in these cases, patient number is highlighted in bold

Patient	SAFE	SAFE_LoC	CRS-*r*	CRS-r_LoC	SAFE > CRS-*r*	CRS-*r* > SAFE
**#1**	5	eMCS	12	MCS+	Visual pursuit; functional object use*	Reproducible movement to command
**#2**	1	MCS-	4	VS/UWS	Visual fixation*	
**#3**	1	MCS-	7	VS/UWS	Visual pursuit*	
#4	5	MCS+	11	MCS+	Visual pursuit; automatic motor response; non-functional intentional communication	
**#5**	7	eMCS	5	VS/UWS	Reproducible movement to command; visual pursuit; automatic motor response; functional communication*	
**#6**	2	MCS+	9	MCS-	Reproducible movement to command*	Visual pursuit
**#7**	9	eMCS	15	MCS+	Reproducible movement to command; object recognition; functional object use*; non-functional intentional communication	
#8	2	MCS-	10	MCS-	Visual fixation	
**#9**	7	MCS+	10	MCS-	Reproducible movement to command*; visual pursuit; intelligible verbalization*; non-functional intentional communication*	
#10	3	MCS-	10	MCS-	Localization to noxious stimulation	
**#11**	1	MCS-	3	VS/UWS	Localization to noxious stimulation*	
#12	9	eMCS	21	eMCS	Object recognition	Consistent movement to command

*Abbreviations* SAFE = Social And Family Evaluation Scale; CRS-r = coma Recovery Scale-revised; LoC = Level of Consciousness; VS/UWS = Vegetative State/Unresponsive Wakefulness Syndrome; MCS -= Minimally Conscious State minus; MCS + = Minimally conscious State plus; eMCS = emerging from Minimally Conscious State

By comparing concordant and discordant cases, no differences emerged in caregivers’ sociodemographic variables (caregiver’s age: U_(20)_ = 54; *p* = .71; caregiver’s education: t_(20)_ = − 0.23; *p* = .81; sex: χ^2^ = 0.02; *p* = .86), hours of care (U_(20)_ = 58; *p* = .92), and patients’ time since injury (U_(20)_ = 48; *p* = .44). Conversely, a significant difference between concordant and discordant cases was found by considering the patients’ diagnosis (χ^2^ = 7.25; *p* = .007). Specifically, the concordant cases were characterized by a higher proportion of patients with VS/UWS diagnosis. In other words, this result suggested that the discordance between SAFE and CRS-r was more frequent when evaluating patients being at least in MCS according to CRS-r.

## Discussion

The preliminary results of the SAFE validation process showed high internal consistency, high criterion validity, and very high test-retest reliability, thus supporting its usefulness in detecting meaningful behaviours that may be unnoticed by clinicians for several reasons. For instance, despite the already known possibility of a late recovery for both VS/UWS and MCS patients [21–27], in some settings, patients with DoC are not re-evaluated over time, thus missing some behavioural signs that, conversely, might be noticed by the informal caregivers informing about possible improvements in the LoC. Moreover, better knowledge of both the patient’s history and preferences makes it easier to interpret some behavioural signs by the informal caregiver as well as select the most appropriate stimuli to elicit the responses [13, 28]. The SAFE makes this possible in a standardised manner and in a directly comparable way to the CRS-r, thus contributing to the adoption of a common language between informal caregivers and expert clinicians which might improve their alliance in taking care of patients with DoC [15] which plays a pivotal role within the pathways of care. Indeed, a previous study showed how different views, expectations and hopes for recovery, divergent goals, and contradictory feelings/thoughts between families and health professionals are known to be potential sources of conflict in decision-making with patients with VS/UWS [29], and one of the proposed key factors to prevent or resolve this ethical and practical conflict is to provide an accurate diagnosis [29].

Despite the SAFE not allowing a clinical diagnosis of patients with DoC, the direct comparability to the CRS-r allowed us to qualitatively compare the behaviours detected across the two scales. The results showed some discrepancies since, in more than half of the sample, the informal caregivers detected higher-level behaviours than those detected by the clinicians, and, in most of these cases, this corresponded to a higher LoC. This result might indicate an overestimating tendency characterizing the informal caregivers as suggested by previous studies [30, 31]. Moreover, the impact of the expert clinician’s scientific knowledge in detecting and interpreting impossible and improbable signs of consciousness should be considered [32]. This could be the case of the clinically diagnosed VS/UWS patient in our study for whom the informal caregiver detected voluntary behavioural signs indicative of a higher LoC as if the patient was eMCS. However, in previous studies, the informal caregivers’ opinions about the LoC of patients with DoC were collected through general questions focusing on the communication and environmental interaction abilities, whilst the SAFE drives informal caregivers to focus on specific behavioural signs that match with those usually considered during clinical evaluations, limiting in this way the possibility that the found discrepancies were due to looking into different elements. Moreover, a further study [33] did not support the hypothesis of a general overestimating tendencies characterizing caregivers of patients with DoC. Indeed, the authors reported that a high proportion of informal caregivers (33 out of 44) assumed the same LoC as detected by clinicians with standard clinical assessments through CRS-r, and, in the remaining cases, a LoC underestimation was reported. For these reasons, on the other hand, one may hypothesize that the discrepancies between the behaviours detected through the CRS-r and SAFE in our study could be due to the better knowledge informal caregivers had of their relatives with DoC and their salience in eliciting a response compared to clinicians. Indeed, the most frequently overestimated behaviours by the SAFE than CRS-r (i.e., visual pursuit and consistent movement to command) belonged to the most sensitive domains to the presence of caregivers (i.e., visual and auditory), in line with previous studies describing differences on patients’ responsiveness between the evaluation performed by clinicians alone and clinicians together with informal caregivers [11–13]. To disentangle these cruces, future studies should differentiate between who administers the sensory stimuli and who scores the patient’s behavioural responses during the evaluation of the LoC (e.g., the informal caregiver provides the stimuli while the clinician scores the patient’s responses, and vice-versa). By adopting this kind of paradigm, it would be possible determining whether the discordance between clinicians and informal caregivers is due to an optimistic interpretation of patients’ behaviours by informal caregivers or increased patients’ responsiveness to salient stimuli provided by informal caregivers. With the same aim, future longitudinal studies should investigate whether the SAFE total score correlates with patients’ clinical evolution, as it happens for the CRS-r total score.

It is worth noticing that the detection of higher-level behaviours by the SAFE than the CRS-r was more frequent in patients clinically showing at least minimal signs of consciousness (8 out of 12), in line with a recent work highlighting the absence of any effect of the presence of informal caregiver during evaluation through CRS-r in VS/UWS patients [12]. The authors explained their results by relying on less sensitivity for salient stimuli characterizing VS/UWS patients [34, 35]. Nevertheless, against this interpretation, in our study, we found discrepancies between SAFE and CRS-r detected behaviours also in some VS/UWS patients. The main reason behind this difference between our and a previous study [12], could be related to the involvement of informal caregivers only for auditory stimulation in the previous study, thus preventing the detection of other behavioural signs such as visual fixation and visual pursuit. Indeed, in the majority of UWS patients for whom discrepancies were found between SAFE and CRS-r in our work, visual fixation and visual pursuit were the main higher-level behaviours detected by the SAFE over the CRS-r.

### Limitations

The main weakness of the present study was represented by the small sample size that requires caution in the generalization of results thus, further work with a larger sample size is needed.

Furthermore, we did not collect any caregivers’ psychological variable that may play a role in overestimating the LoC of patients with DoC as suggested by previous works [30, 36, 37]. Specifically, Suppes and Fins [36] highlighted that informal caregivers’ emotional entanglement with patients led to judge the LoC and the recovery probability as higher than what was done by clinicians. Moreover, the overestimation of LoC may strongly depend on caregivers’ psychological distress as shown by Moretta et al. [30] who highlighted that caregivers overestimating the patients’ LoC were characterized by higher levels of depressive symptoms and worries about the future. For this reason, in the near future, caregivers’ psychological features should be taken into account when considering their opinions about the LoC of patients with DoC.

## Conclusion

Overall, the present work provided promising preliminary data for the adoption of the SAFE scale to collect in a standardised way the opinions of the informal caregivers about the LoC of patients with DoC. This is in line with both international guidelines recommendations [14] and scientific literature suggestions [11, 13, 16, 30, 37] about the need for caregivers’ involvement to better frame the actual LoC of patients with DoC due to their better knowledge of patients and the higher probability they have in eliciting patients’ behavioural responses. The use of the SAFE is complementary to and cannot replace the adoption of the CRS-r which remains the gold-standard to detect the patients’ LoC and make an accurate clinical diagnosis. Nevertheless, the SAFE could be adopted in home settings, as part of telemedicine monitoring, and in long-term care settings to empower caregivers in their caring activities and to inform clinicians about the need to re-evaluate the patient with DoC due to possible changes in the LoC. Finally, by providing a standardised assessment, SAFE could be an important tool for monitoring and avoiding misalignment between clinicians and family members in assessing consciousness also during the early stages of rehabilitation.

## Electronic supplementary material

Below is the link to the electronic supplementary material.

Supplementary Material 1

## Data Availability

Data will be available on request.
