# Supplementary material for: The social and family evaluation (SAFE) scale for caregivers of individuals with disorders of consciousness: preliminary results
Source: Neurol Sci. 2024 Jul 27;46(1):393–400. doi: 10.1007/s10072-024-07685-4 (PMC11698764; doi:10.1007/s10072-024-07685-4)
Supplement: Supplementary file 1 — Supplementary Material 1 [file 10072_2024_7685_MOESM1_ESM.docx]

**Supplementary Information for the article titled:**

*The social and family evaluation (SAFE) scale for caregivers of individuals with disorders of consciousness: preliminary results*

**Authors**

Magnani F.G.^1^, Cacciatore M.^1^, Barbadoro F.^1, *^, Ippoliti C.^1^, Sattin D.^2^, Magliacano A.^3^, Draghi F.^3^, De Nisco A^3^, Hakiki B.^3,4^, Cecchi F,^3,4^, Spinola M.^3^, Estraneo A.^3^, Leonardi M.^1^

**Affiliations**

^1^ SC Neurologia, Salute Pubblica, Disabilità; Fondazione IRCCS Istituto Neurologico Carlo Besta, Milan (Italy)

^2^ Istituti Clinici Scientifici Maugeri IRCCS, Health Directorate, Via Camaldoli 64, 20138 Milan, Italy;

^3^IRCCS Fondazione Don Carlo Gnocchi ONLUS, Florence (Italy)

^4^ Department of Experimental and Clinical Medicine, University of Florence, Firenze, Italy

*^*^Correspondence to*: Filippo Barbadoro; e-mail: [filippo.barbadoro@istituto-besta.it](mailto:filippo.barbadoro@istituto-besta.it)

**SAFE administration and structure description**

In what follows, the Italian version of the Social And Family Evaluation (SAFE) scale is provided. Specifically, the SAFE firstly requires the collection of both caregiver- and patient-related socio-demographical and clinical information including age, sex, and education, hours of care spent on average in a week by the caregiver, patient’s time since injury, aetiology, and the last clinical diagnosis received. Then, the instructions are provided in written form along with a deep explanation by the clinician. Specifically, the written instructions inform about the general structure of the SAFE and the answer modality: across the SAFE, the caregiver finds the description of different behaviours characterized by different difficulty levels. It is asked to indicate, in the caregiver’s opinion, the level of functioning that best corresponds to the current state of the patient (by referring to the last 15 days) for each of the reported behaviour answering to YES/NO questions (fourth and fifth column, respectively in the SAFE scale provided below). For instance, the caregiver is asked to think if the patient is able to look at an object that rapidly moves in front of his/her eyes (TRIAL; second column in the SAFE scale provided below) at least 2 times out of 4 total trials (CRITERION; third column in the SAFE scale provided below). It is further explained that there are no right or wrong answers, and the enlisted behaviours have different levels of difficulty, so that it is not a problem if the caregiver thinks that the patient is not able to perform a certain behaviour. Furthermore, the caregiver is allowed to write any annotations of interest concerning the specific behaviour considered (sixth column in the SAFE scale provided below). Finally, the last column of the SAFE scale has to be completed by the professional who will convert the YES/NO answers provided by the caregiver into 1/0 scores to allow the total score computation. As already explained in a previous work (Sattin et al., 2022), the behaviours are arranged across the SAFE in 5 different subscales addressing visual, sensory-motor, language-communication, pain perception, and task execution (both object-related and not related) abilities.

**The Social And Family Evaluation scale for caregivers of patients with DoC – Italian version**

**SAFE**

| ***Dati di chi compila il questionario (caregiver):*** | | | | | | | | |
| --- | --- | --- | --- | --- | --- | --- | --- | --- |
| Nome | | | | | Cognome | | | |
| Età | Data di nascita __/__/____ | | Genere M F | | Anni di studio ___ | Data di compilazione __/__/____ | | |
| Quante ore alla settimana, mediamente, presta assistenza alla persona con diagnosi di DoC? N° ore ____ | | | | | | | | |
| ***Dati della persona con diagnosi di DoC:*** | | | | | | | | |
| Nome | | | | Cognome | | | | |
| Data di nascita __/__/____ | | Genere M F | | Anni di studio ___ | Data dell’evento acuto __/__/____ | | Eziologia | Diagnosi |

**Istruzioni**

*Gentile* [Nome caregiver] ____________,

*di seguito troverà descritte delle possibili prove, con un differente livello di difficoltà, che potrebbero essere somministrate a* [Nome paziente] ____________.

*Quello che le viene chiesto nel presente questionario è di indicare, a suo parere, il livello di funzionamento che corrisponde maggiormente allo stato attuale del suo assistito per ognuna delle prove riportate.*

*In particolare, le stiamo chiedendo di rispondere mettendo una X nella colonna SI o in quella NO se, secondo lei,* [Nome paziente] *____________sarebbe o non sarebbe in grado di eseguire la prova prevista in ciascun compito (riportata nella colonna “PROVE”) per almeno un numero di volte pari al valore riportato nella colonna “CRITERIO”.*

*Per esempio, se la PROVA a cui si chiede di pensare riguarda la capacità di guardare un oggetto che viene spostato rapidamente davanti agli occhi di* [Nome paziente] *____________ per almeno 2 volte su 4 prove totali (CRITERIO), lei dovrà rispondere SI o NO in base a quanto lei pensa che* [Nome paziente] *_____________sia in grado di fare “almeno due volte” quel movimento con gli occhi. Non esistono risposte giuste o sbagliate e come vedrà le possibili prove hanno diversi livelli di difficoltà. Non si preoccupi nel caso in cui pensa che* [Nome paziente] ____________ *non sia in grado di eseguire una determinata prova; anche in questo caso le chiediamo di rispondere indicando NO nella specifica colonna. Pertanto, le stiamo chiedendo di darci la sua opinione alla luce della sua esperienza come caregiver in merito a come risponderebbe il suo assistito facendo riferimento agli ultimi 15 giorni.*

|  | **PROVE** | **CRITERIO**  **Secondo lei *[nome paziente]***  ***Negli ultimi 15 giorni*** | **SI** | **NO** | **note** | **Punteggio (da compilare a cura del professionista)** |
| --- | --- | --- | --- | --- | --- | --- |
| **FUNZIONE VISIVA** | |  |  |  |  |  |
| Provi a pensare se *[nome paziente]* sarebbe in grado di | *indirizzare* lo sguardo per fissare un oggetto (es. uno specchio, un cellulare, una foto, ecc.) che viene posto in alto, in basso, a destra, a sinistra rispetto ai suoi occhi. | immaginando di aver fatto 4 prove, sarebbe stato in grado di fissare l’oggetto *almeno 2 volte verso una qualsiasi direzione*? |  |  |  |  |
| Provi a pensare se *[nome paziente]* sarebbe in grado di | *seguire* con gli occhi degli oggetti o delle persone che si muovono intorno a lui in una qualsiasi direzione senza perdere la mira. | immaginando di aver fatto 8 prove (2 verso l’alto, 2 verso il basso, 2 verso destra, 2 verso sinistra), sarebbe stato in grado di seguire persone/oggetti che si muovono *almeno 2 volte*? |  |  |  |  |
|  |  |  |  |  |  |  |
| **FUNZIONI SENSORI-MOTORIO** | |  |  |  |  |  |
| Provi a pensare se *[nome paziente]* sarebbe in grado di | *afferrare* un oggetto (ad esempio una palla da tennis) se messo sul dorso della sua mano e fatto rotolare verso il dito indice ed il pollice. | Immaginando di fare 4 prove, sarebbe stato in grado di afferrare l’oggetto *almeno 3 volte* (ad esempio aprendo le dita e ruotando in polso come per afferrare l’oggetto)? |  |  |  |  |
| Provi a pensare se *[nome paziente]* sarebbe in grado di | *eseguire* movimenti spontanei, cioè senza che ci sia una richiesta specifica e finalizzati a qualcosa, come grattarsi, afferrare le sbarre del letto, aprire la bocca se ha vicino un cucchiaio, salutare con la mano, ecc (non considerare sbadigli o movimenti riflessi della bocca) | sarebbe stato in grado di eseguire *almeno 2 movimenti spontanei*? |  |  |  |  |
|  |  |  |  |  |  |  |
| **LINGUAGGIO – COMUNICAZIONE** | |  |  |  |  |  |
| Provi a pensare se *[nome paziente]* sarebbe in grado di | *pronunciare* delle parole semplici (parole che siano composte almeno da tre lettere (consonante-vocale-consonante o viceversa), come per esempio: mam, ama, per, ecc.) | sarebbe stato in grado di pronunciare *almeno 2 parole semplici*? |  |  |  |  |
| Provi a pensare se *[nome paziente]* sarebbe in grado di | *esprimere “SI” e “NO”* a voce o muovendo la testa su e giù/a destra e a sinistra, o con le mani/le dita, o muovendo gli occhi o le palpebre (o in qualsiasi altro modo) in risposta a domande semplici?  Il “SI” e il “NO” devono essere espressi in modo corretto a seconda della richiesta.  Ad esempio:  rispondere “SI” alla domanda:  *mi sto toccando il naso?*  O  *sto battendo le mani?*  mentre realmente ci si tocca il naso o si battano le mani  E  rispondere NO alla stessa domanda:  *mi sto toccando il naso?*  O  *sto battendo le mani?*  Mentre NON ci si tocca il naso o non si battano le mani | Immaginando di fare 6 prove (3 che richiedano risposta “SI”, 3 che richiedano risposta “NO”), sarebbe stato in grado di rispondere “SI” o “NO” in maniera corretta *almeno 2 volte*? |  |  |  |  |
|  |  | Se la risposta è “SI” alla domanda precedente, secondo lei *[nome paziente]* sarebbe stato in grado di rispondere “SI” e “NO”  in maniera corretta a *tutte e 6 le prove*? |  |  |  |  |
| **DOLORE** |  |  |  |  |  |  |
| Provi a pensare se *[nome paziente]* sarebbe in grado di | *muovere* il braccio (o la gamba) per andare a toccare una parte del corpo sulla quale è stato applicato uno stimolo doloroso.  Ad esempio:  se venisse punta o pizzicata la mano destra, sarebbe in grado di muovere il braccio sinistro verso la mano che è stata punta (anche senza raggiungere esattamente il punto stimolato)? | immaginando di applicare lo stimolo 4 volte, sarebbe stato in grado di muovere il braccio (o la gamba) come indicato *almeno 2 volte*? |  |  |  |  |
|  |  |  |  |  |  |  |
| **ESECUZIONE DI COMPITI** |  |  |  |  |  |  |
| COMPITI da eseguire con gli occhi o altre parti del corpo senza utilizzare oggetti: | |  |  |  |  |  |
| Provi a pensare se *[nome paziente]* sarebbe in grado di | *guardare* in alto oppure in basso, guardare a destra oppure a sinistra, se glielo chiede  oppure  toccare con le mani qualcosa (per esempio, la sua mano, il suo naso o altre parti del corpo), se glielo chiede  oppure  fare dei movimenti con la bocca (per esempio, tirare fuori la lingua, aprire o chiudere la bocca, dire “ah”), se glielo chiede | immaginando di fare 4 prove, sarebbe stato in grado di eseguire *almeno 3 movimenti* richiesti? |  |  |  |  |
|  |  | Se la risposta è “SI” alla domanda precedente, secondo lei *[nome paziente]* sarebbe stato in grado di eseguire correttamente i movimenti richiesti in *tutte e 4 le prove*? |  |  |  |  |
| COMPITI da eseguire con gli occhi utilizzando oggetti: | |  |  |  |  |  |
| Provi a pensare se *[nome paziente]* sarebbe in grado di | *riconoscere* oggetti familiari e di uso quotidiano (come, ad esempio, il pettine e lo spazzolino), pensando di fare 4 prove come in questo esempio:  Prova 1  Presentare, per esempio, uno spazzolino alla destra di *[nome paziente]* e una palla alla sua sinistra e chiedere di guardare la palla  Prova 2  Scambiare la posizione degli oggetti senza farsi vedere (dietro la schiena) e chiedere nuovamente di guardare la palla  Prove 3 e 4  Chiedere di guardare lo spazzolino ripetendo le prove 1 e 2 | immaginando di fare tutte e 4 le prove, sarebbe stato in grado di guardare correttamente l’oggetto da lei richiesto di volta in volta per *almeno 3 volte*? |  |  |  |  |
|  |  | Se la risposta è “SI” alla domanda precedente, secondo lei *[nome paziente]* sarebbe stato in grado di guardare correttamente l’oggetto da lei richiesto in *tutte e 4 le prove*? |  |  |  |  |
| COMPITI da eseguire con diverse parti del corpo utilizzando oggetti: | |  |  |  |  |  |
| Provi a pensare se *[nome paziente]* sarebbe in grado di | *Muovere* un braccio o una gamba verso un oggetto (ad esempio un pettine o una palla) ben visibile come per andarlo a toccare se glielo si chiede.  Ad esempio:  Prova 1  Mostrare la palla vicino alla gamba di destra e chiedere “tocca la palla”  Prova 2  Mostrare la palla vicino alla gamba di sinistra e chiedere “tocca la palla”  Prova 3  Mostrare la palla vicino alla gamba di sinistra e chiedere “tocca la palla”  Prova 4  Mostrare la palla vicino alla gamba di destra e chiedere “tocca la palla”  Le stesse prove possono essere fatte per le braccia utilizzando il pettine, nel caso in cui *[nome paziente]* non riesca a muovere le gambe | immaginando di fare tutte e 4 le prove, sarebbe stato in grado di muovere il braccio/la gamba verso l’oggetto da lei richiesto per *almeno 3 volte*? |  |  |  |  |
| COMPITI da eseguire con le mani utilizzando oggetti: | |  |  |  |  |  |
| Provi a pensare se *[nome paziente]* sarebbe in grado di | *Mostrare correttamente l’uso* di oggetti familiari di uso comune (ad esempio mostrando un cellulare e una tazza) se gli viene chiesto di eseguire il corretto movimento con le mani (ad esempio portandosi il cellulare all’orecchio o la tazza alla bocca). | sarebbe stato in grado di mostrare  *almeno 2 volte* l’uso di ciascun oggetto? |  |  |  |  |
|  | | | | | | |
|  |  |  |  |  |  | Totale ____ |

**Table S1** The table shows the total number of times each non-reflexive behaviour (first column) was detected by the CRS-r over the SAFE (second column) and *vice-versa* (third column).

| **Target behaviour** | **CRS-r > SAFE** | **SAFE > CRS-r** |
| --- | --- | --- |
| Consistent movement to command | 1 | 0 |
| Reproducible movement to command | 1 | 4 |
| Object recognition | 0 | 2 |
| Object localization | 0 | 0 |
| Visual pursuit | 1 | 5 |
| Visual fixation | 0 | 2 |
| Functional object use | 0 | 1 |
| Automatic motor response | 0 | 2 |
| Object manipulation | 0 | 0 |
| Localisation to noxious stimulation | 0 | 2 |
| Intelligible verbalization | 0 | 1 |
| Functional accurate communication | 0 | 2 |
| Non-functional intentional communication | 0 | 3 |

**References**

Sattin D, Magnani FG, Cacciatore M, Leonardi M. Towards a New Assessment Tool for Caregivers of Patients with Disorders of Consciousness: The Social and Family Evaluation Scale (SAFE). Brain Sci. 2022 Feb 28;12(3):323. doi: 10.3390/brainsci12030323. PMID: 35326279; PMCID: PMC8946058.
